# Comparison between immunotherapy efficacy in early non-small cell lung cancer and advanced non-small cell lung cancer: a systematic review

**DOI:** 10.1186/s12916-022-02580-1

**Published:** 2022-11-07

**Authors:** Yimin Wang, Chuling Li, Zimu Wang, Zhaofeng Wang, Ranpu Wu, Ying Wu, Yong Song, Hongbing Liu

**Affiliations:** 1 Department of Respiratory Medicine, Jinling Hospital, Nanjing Medical University, 305 East Zhongshan Road, Nanjing, China; 2 Department of Respiratory Medicine, Jinling Hospital, Nanjing University School of Medicine, 305 East Zhongshan Road, Nanjing, China; 3 Department of Respiratory Medicine, Jinling Hospital, Southeast University School of Medicine, Nanjing, China; 4 Department of Respiratory Medicine, Zhongda Hospital, School of Medicine, Southeast University, 87 Dingjiaqiao, Nanjing, China

**Keywords:** Immunotherapy, Non-small cell lung cancer, Systematic review

## Abstract

**Background:**

Currently, immunotherapy is widely used in the treatment of various stages of non-small cell lung cancer. According to clinical experience and results of previous studies, immunotherapy as neoadjuvant therapy seems to exhibit better efficacy against early resectable non-small cell lung cancer as compared to advanced lung cancer, which is often defined as unresectable non-small cell lung cancer. However, this observation has not been established in clinical studies. This systematic review aimed to evaluate the efficacy of immunotherapy in early and late lung cancer, wherein objective response rate (ORR) and disease control rate (DCR) were used as evaluation indexes. The present study also evaluated the safety of immunotherapy in early and late lung cancer, wherein the rate of treatment-related adverse reactions (TRAEs) was used as an indicator.

**Methods:**

Electronica databases, including PubMed, Cochrane Library, Embase, and other databases, were searched to identify relevant studies. Besides this, all the available reviews, abstracts, and meeting reports from the main international lung cancer meetings were searched manually. ORR, DCR, and TRAEs were extracted as the primary outcomes.

**Results:**

A total of 52 randomized controlled trials involving 13,660 patients were shortlisted. It was observed that immunotherapy alone significantly improved DCR in early lung cancer in comparison to advanced lung cancer. Importantly, the improvement in ORR was not to the same extent as reported in the case of advanced lung cancer. The combination of immunotherapy with other therapies, especially immunochemotherapy, significantly improved ORR and DCR in early lung cancer. In terms of safety, immunotherapy either alone or in combination with other therapies exhibited a better safety profile in early lung cancer than in advanced lung cancer.

**Conclusions:**

Altogether, the benefits of immunotherapy in early lung cancer appeared to be better than those observed in advanced lung cancer, especially with the regard to the regimen of immunotherapy in combination with chemotherapy. In terms of safety, both immunotherapy alone and its combination with chemotherapy were found to be safer in early lung cancer as compared to advanced lung cancer.

**Supplementary Information:**

The online version contains supplementary material available at 10.1186/s12916-022-02580-1.

## Background

Lung cancer is still among the most common cancer types reported worldwide. In fact, it is the most common cause of cancer-related deaths [1]. Importantly, 80% of the newly discovered lung cancers are contributed by non-small cell lung cancer (NSCLC). A large number of studies are being conducted to explore therapeutic strategies for NSCLC [2]. The emergence of targeted therapy enabled the treatment of patients with NSCLC tested positive for driver gene. Importantly, targeted therapy prolonged the survival in such patients. In the case of patients with driver gene negative profiles, only chemotherapy has been used for a long time [3]. Following this, immunotherapy emerged as a promising approach. Previous clinical studies on immunotherapy showed that immunotherapy incurred better clinical benefits than previously used chemotherapeutic strategies. Importantly, these benefits were observed when it was used alone with PD-1/PD-L1 inhibitors, CTLA-4 inhibitors, or in combination with chemotherapy, antivascular drugs, or even in the case of dual immunotherapy agents [4–14].

Currently, immunotherapy is widely used for the treatment of various stages of NSCLC. Previous clinical studies confirmed the efficacy of immunotherapy in advanced NSCLC [5, 15, 16]. In fact, more and more clinical studies are being conducted to assess the efficacy of immunotherapy in NSCLC in a detailed manner [17–19]. In general, immunotherapy has prolonged the survival of patients with advanced NSCLC. However, there are cases where patients did not benefit significantly. In fact, the incidence of treatment-related adverse reactions remains high. Importantly, such incidences of adverse reactions are much lower than those reported in the case of chemotherapy. For early resectable NSCLC, single-arm trials and meta-analyses previously reported that patients exhibited good pathological responses following neoadjuvant immunotherapy. Several randomized controlled trials that were reported at the conference in the current year showed that neoadjuvant immunotherapy achieved significant efficacy in early resectable NSCLC, with a low incidence of treatment-related adverse events [20, 21].

Clinical cases and existing clinical research data showed that immunotherapy as a new adjuvant treatment incurred better curative effects in the case of resectable lung cancer as compared to late lung cancer. However, none of the currently available studies have established whether the application of immunotherapy in the early resection of lung cancer is beneficial or its application in advanced unresectable lung cancer.

In the present study, ORR and DCR were used as evaluation indexes/indices to assess the efficacy of immunotherapy in early and late lung cancer, while the rate of adverse reactions was used as an indicator to evaluate the safety of immunotherapy in early and late lung cancer.

## Methods

### Data source and searches

We searched all RCTs related to NSCLC from the PubMed, Embase, Cochrane Library, and other databases from inception until November 2021, with no start data limit, applied. In addition, we also conducted a manual search for all available reviews, abstracts, and meeting reports from the main international lung cancer meetings. The search keywords included “non-small cell lung cancer,” “early lung cancer,” “early stage of lung cancer,” “advanced lung cancer,” “immunotherapy,” “PD-1 inhibitor,” “PD-L1 inhibitor,” “programmed cell death-ligand 1,” “CTLA-4 inhibitor,” “cytotoxic T-lymphocyte antigen-4 inhibitor,” and others (Additional file 1: Supplemental Methods). The language the RCTs used was limited to English. Two authors accomplished the search independently, and any discrepancy was resolved through mutual discussion to reach a final consensus.

### Selection criteria

According to the PICOS framework, papers that conformed to the following criteria were included: (I) only early non-small lung cancer patients or advanced non-small lung cancer patients; (II) papers involving an immunotherapy cohort and either immunotherapy alone cohort or immunotherapy combined with other therapies cohort; (III) papers that reported the outcomes included more than one of the following: ORR, DCR, and TRAEs; and (IV) papers that included all RCTs and multicenter single-arm studies. Case-control studies, retrospective studies, cohort studies, case reports, meta-analyses, and systematic reviews were excluded.

### Data extraction and risk of bias assessment

The authors independently reviewed and evaluated the title, abstract, full text, and supplementary materials of the included studies and extracted all the data. All extracted data were tabulated including the country clinical trial number, publication date, first author, intervention, and the number of participants in each intervention group. In addition, data on the complete response (CR), partial response (PR), stable disease (SD), ORR, DCR, and TRAEs were also included. The risk ratio (RR) and the corresponding 95% CIs for ORR and DCR were also extracted. Items not reported in the included studies were represented by NR (not reported).

We applied The Cochrane Collaboration's Risk of Bias tool to evaluate the quality of the included articles. The evaluation factors included randomness, double-blindness, the integrity of the outcome data, and bias in selective reporting, among others. The risk of bias was assessed with reference to the following criteria: low risk, high risk, and ambiguous risk. Two authors independently completed the quality evaluation of the extraction of the review data for the included studies. Finally, the controversial portion was resolved through active discussion [22].

### Data synthesis and analysis

ORR and DCR were considered as the primary endpoints in this systematic review. A Bayesian approach was accordingly adopted. ORR and DCR were treated as dichotomous variables; therefore, risk ratios (RRs) were applied to present these parameters. We applied the *χ*^2^ test and *I*^2^ statistics to evaluate the statistical heterogeneity of the included studies. A fixed-effects model was selected to count the pooled estimate when the *p*-value for *χ*^2^ > 0.1 and *I*^2^ was < 50%. Otherwise, a random-effects model was applied to combine the studies. At *I*^2^ statistic > 50% or *P*-value for *χ*^2^ < 0.1, the values were considered to be statistically significant for heterogeneity [23]. Subgroup analyses were conducted according to the treatment regimens across the entire cohort. Cohorts of immunotherapy alone or immunotherapy combined with other therapies were categorized for subgroup analyses. Based on the prearranged grouping factors, we collected the data of relevant subgroups in all included trials. We then applied the funnel plot and Egger's test and Begg's test to evaluate publication bias.

Stata v15.1 (Stata Corporation, College Station, TX, USA) was applied to perform all statistical analyses. *P*-values were two-sided and considered to be statistically significant, except for *P* < 0.05.

## Results

### Systematic review and characteristics

The present study initially identified a total of 4747 studies. Among these, 925 studies were excluded due to duplication. Finally, a total of 52 studies were included, following a screening of abstracts and full texts according to the selection criteria. The research selection process followed in the present study is shown in Additional file 1: Fig. S1. The main features of the included studies are shown in Table 1.

**Table 1:** Primary characteristics and the results of the applicable studies

Study	Year	Treatment	ORR		DCR		TRAE	Grade ≥ 3 TRAEs
			No of response	No of patients	No of response	No of patients		
RCTs of early-staged lung cancer								
checkmate159 [24]	2021	nivo	2	22	20	22	23%	4.5%
IFCT-1601 IONESCO [25]	2017	durva	4	43	37	43	NR	NR
NADIM [26]	2020	nivo+chemo	36	46	44	46	93%	30%
NEOSTAR [27]	2020	nivo	5	23	NR	23	NR	13%
		nivo+ipi	4	21	NR	21	NR	10%
SAKK16 14 [21]	2021	durva+chemo	36	62	52	62	NR	88.1%
PRINCEPS	2020	atezo	16	29	NR	29	NR	NR
Checkmate-816	2021	nivo+chemo	NR	NR	NR	NR	41%	11%
IMpower010 [20]	2021	atezo+chemo	NR	NR	NR	NR	93%	22%
Lcmc3	2021	atezo	NR	NR	NR	NR	67%	16%
RCTs of advanced lung cancer								
KEYNOTE-001 [28]	2019	perm	143	550	490	550	71%	12%
KEYNOTE-010 [29]	2015	perm-2mg/kg	62	344	NR	344	63%	13%
		perm-10mg/kg	64	346	NR	346	66%	16%
KEYNOTE-021 [14]	2016	perm	33	60	53	60	94%	40%
KEYNOTE-024 [30]	2016	perm	69	154	NR	154	73.4%	26.6%
KEYNOTE-042 [31]	2019	perm	174	637	422	637	63%	18%
CheckMate-026 [15]	2017	nivo	55	211	135	211	71%	18%
IMpower110 [7]	2010	atezo	81	277	NR	277	90.2%	30.1%
OAK [11]	2016	atezo	108	622	208	425	64%	15%
poplar [10]	2016	atezo	96	144	NR	144	67%	40%
IMpower130 [9]	2019	atezo+chemo	220	447	256	447	96.1%	74.8%
IMpower131 [8]	2020	atezo+chemo	170	342	277	342	94.6%	69.2%
IMpower132 [5]	2020	atezo+chemo	137	292	NR	292	91.4%	58.4%
KEYNOTE-189 [32]	2020	atezo+chemo	195	410	347	410	99.8%	67.2%
KEYNOTE-407 [6]	2020	atezo+chemo	174	278	239	278	98.6%	74.1%
Camel [13]	2020	camre	111	205	NR	205	99.5%	68.8%
Camel-sq	2021	camre	125	193	170	193	100%	80.3%
Empower-lung1 [4]	2021	cemip	111	283	187	283	57%	14%
JAVELIN Lung 200 [12]	2018	ave	50	264	136	264	64%	10%
MYSTIC [33]	2020	durva	66	286	NR	286	54.2%	14.9%
		durva+chemo	65	268	NR	268	60.1%	22.9%
ORIENT-11 [17]	2020	sinti+chemo	138	266	NR	266	99.6%	61.7%
ORIENT-12 [34]	2021	sinti+chemo	80	179	NR	179	100%	86.6%
RATIONAL-304 [19]	2021	tisle	128	233	NR	233	100%	33.3%
RATIONAL-307 [18]	2021	tisle+pc	87	120	105	120	99.2%	85.8%
		tisle+nab-pc	89	119	108	119	99.2%	83.9%
CheckMate 012 [35]	2017	nivo+ipi(12w)	18	38	30	38	82%	37%
		nivo+ipi (6w)	15	39	22	39	71%	33%
CheckMate 017 [36]	2015	nivo	27	135	66	135	58%	7%
CheckMate 057 [37]	2015	nivo	56	292	130	292	69%	10%
KEYNOTE-025 [16]	2018	perm	8	37	20	37	87%	29%
CheckMate 063 [38]	2015	nivo	17	117	47	117	74%	17%
CheckMate 078 [39]	2018	nivo	56	338	177	338	64%	10%
IMpower150 [40]	2019	atez+beva+chemo	224	397	335	397	NR	67%
		atez+chemo	163	401	317	401	NR	61%
ARCTIC [41]	2020	durva	22	62		62	96.8%	40.3%
CASPLAN [42]	2019	durva	182	268	202	268	98%	62%
PROLUNG [43]	2020	perm+chemo	17	40	28	40	NR	NR
CheckMate 9LA [44]	2021	nivo+ipi+chemo	138	361	302	361	91%	47%
GARNET	2021	dostar	18	67	NR	67	NR	NR
CHOICE-01	2021	toripa+chemo	196	309	NR	309	NR	NR
GEMSTONE-302	2021	sugema+chemo	203	320	NR	320	NR	64.10%
POSEIDON	2021	durva+chemo	137	338	NR	338	NR	44.60%
		durva+treme+chemo	130	338	NR	338	NR	51.80%
RATIONAL-303	2021	tisle	91	423	NR	423	NR	39%
CITYCYPE	2021	toripa+atezo	21	67	NR	67	NR	48%
		atezo	11	68	NR	68	NR	44%
AFT-16	2021	atezo+chemo+cCRT	19	62	48	62	87.1%	NR
PACIFIC [45]	2019	durva	126	443	359	443	96.8%	29.9%
KEYNOTE-598	2021	pem	129	284	202	284	68.3%	19.6%
		pem+ipi	129	284	199	284	76.2%	35.1%

*nivo* nivolumab, *chemo* chemotherapy, *durva* durvalumab, *atezo* atezolizumab, *perm* pembrolizumab, *ave* avelumab, *camre* camrelizumab, *cemip* cemiplimab, *tisle* tislelizumab, *sinti* sintilimab, *ipi* ipilimumab, *pc* paclitaxel and carboplatin, *nab* nanoparticle albumin-bound, *beva* bevacizumab, *treme* tremelimumab, *cCRT* concurrent chemoradiation therapy, *dostar* dostarlimab, *toripa* toripalimab, *sugema* sugemalimab

Altogether, a total of 52 studies were included in the present study. Among these, 43 studies focused on advanced lung cancer, while nine studies focused on early-stage lung cancer. Among the studies related to advanced lung cancer, 27 studies reported the use of immunotherapy alone, whereas 19 studies reported the use of immunotherapy in combination with other treatment strategies. Among these 19 studies on combined therapy, 13 studies explored its combination with chemotherapy, three studies reported immunodouble-drug usage, one study reported a combination of double immunotherapy with chemotherapy, and two studies reported a combination of immunotherapy with chemoradiotherapy. In the case of studies involving early-stage lung cancer, five studies reported immunotherapy alone and five studies reported a combination of immunotherapy with other treatment strategies. For the studies reporting application of immunotherapy combined with other treatment strategies, four studies reported its combination with chemotherapy, while one study reported application of immunodouble-drug.

### ORR

**Fig. 1:**
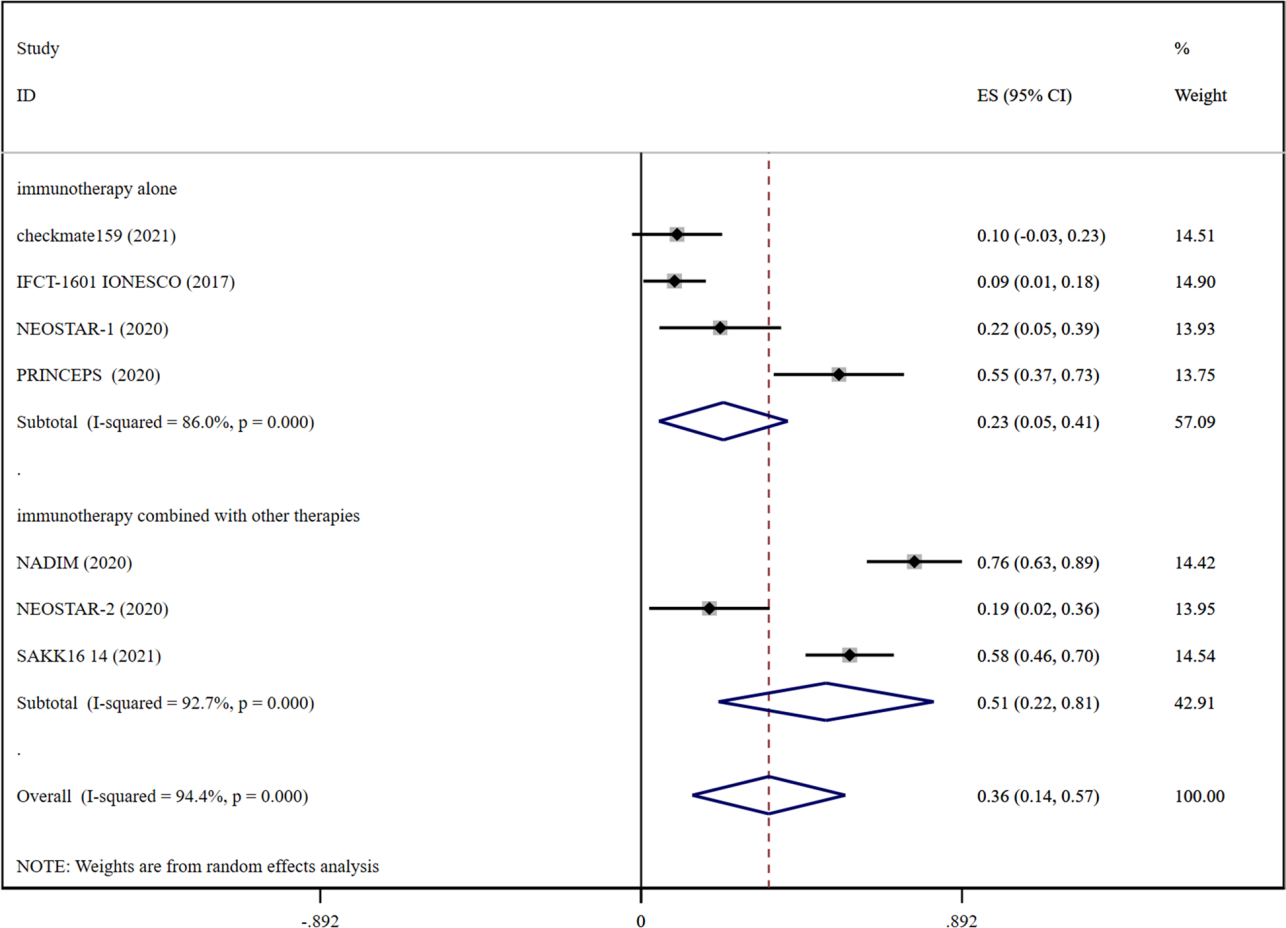
Forest plots presenting pooled ORR risk ratio analysis in early-stage lung cancer and ORR risk ratio analysis for the cohort of immunotherapy alone and immunotherapy combined with other therapies

**Fig. 2:**
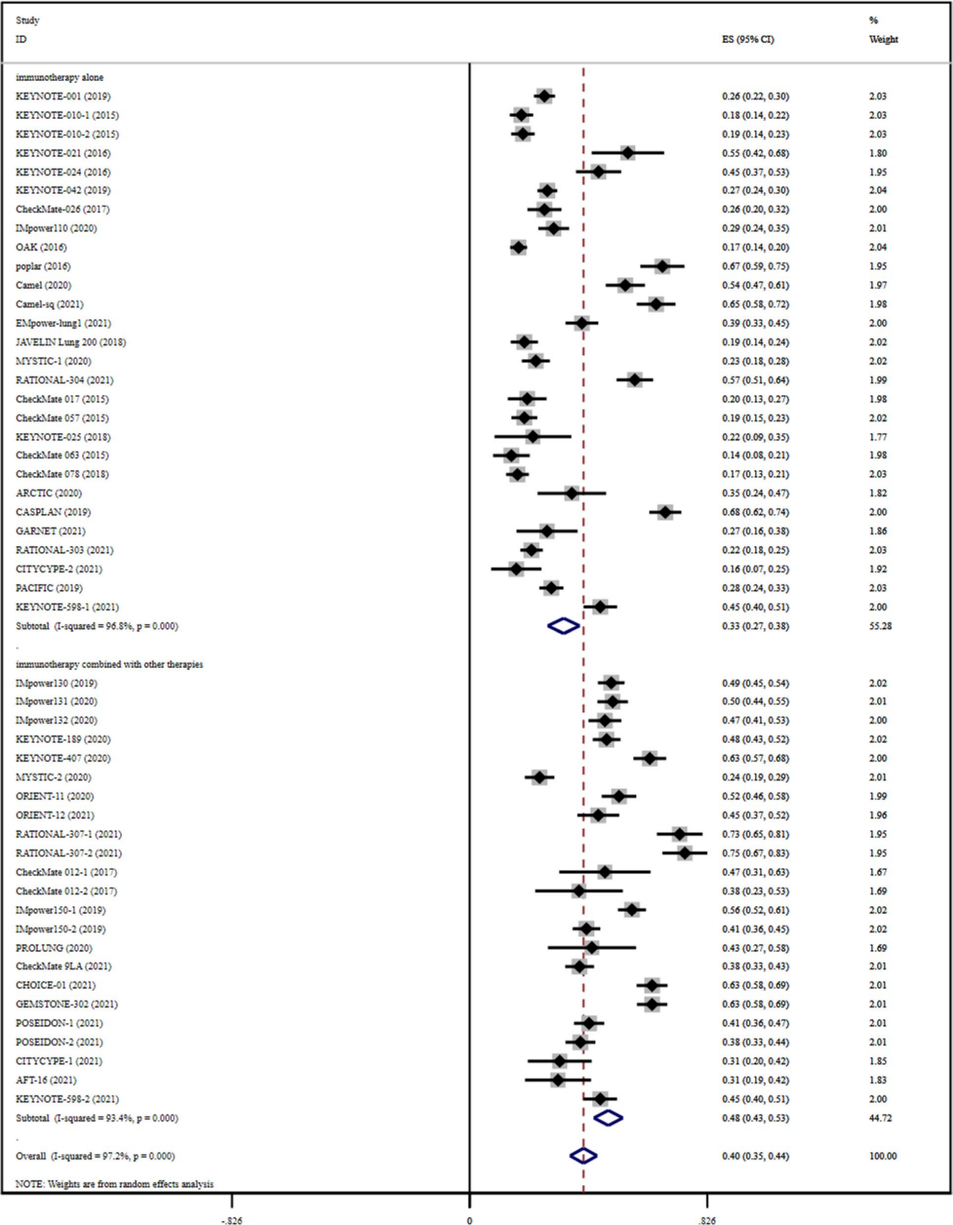
Forest plots presenting pooled ORR risk ratio analysis in advanced lung cancer and ORR risk ratio analysis for the cohort of immunotherapy alone and immunotherapy combined with other therapies

The pooled RR for ORR was recorded to be 0.36 (0.14–0.57) and 0.40 (0.36–0.45) in the case of early-stage lung cancer (Fig. 1) and advanced lung cancer (Fig. 2), respectively. These results showed that immunotherapy incurred a slightly better effect in advanced lung cancer; however, the difference recorded between these two groups was statistically insignificant.

For the immunotherapy alone cohort, pooled RR for ORR was recorded to be 0.23 (0.05–0.41) in early-stage lung cancer (Fig. 1) and 0.33 (0.27–0.38) in advanced lung cancer (Fig. 2). In terms of ORR results, immunotherapy alone exhibited a better effect in advanced lung cancer.

In the case of the cohort for a combination of immunotherapy with other therapies, pooled RR for ORR was recorded to be 0.51 (0.22–0.81) in early-stage lung cancer (Fig. 1) and 0.48 (0.43–0.53) in advanced lung cancer (Fig. 2). These results indicated that the benefit of immunotherapy in combination with other therapies was not significant in the case of early-stage lung cancer when compared with advanced lung cancer.

In cohort for a combination of immunotherapy with chemotherapy, pooled RR for ORR were 0.67 (0.49–0.84) in early-stage lung cancer (Additional file 1: Fig. S2) and 0.52 (0.46–0.58) in advanced lung cancer (Additional file 1: Fig. S3), which indicated that the combination of immunotherapy with chemotherapy regimen was more beneficial in early-stage lung cancer.

### DCR

**Fig. 3:**
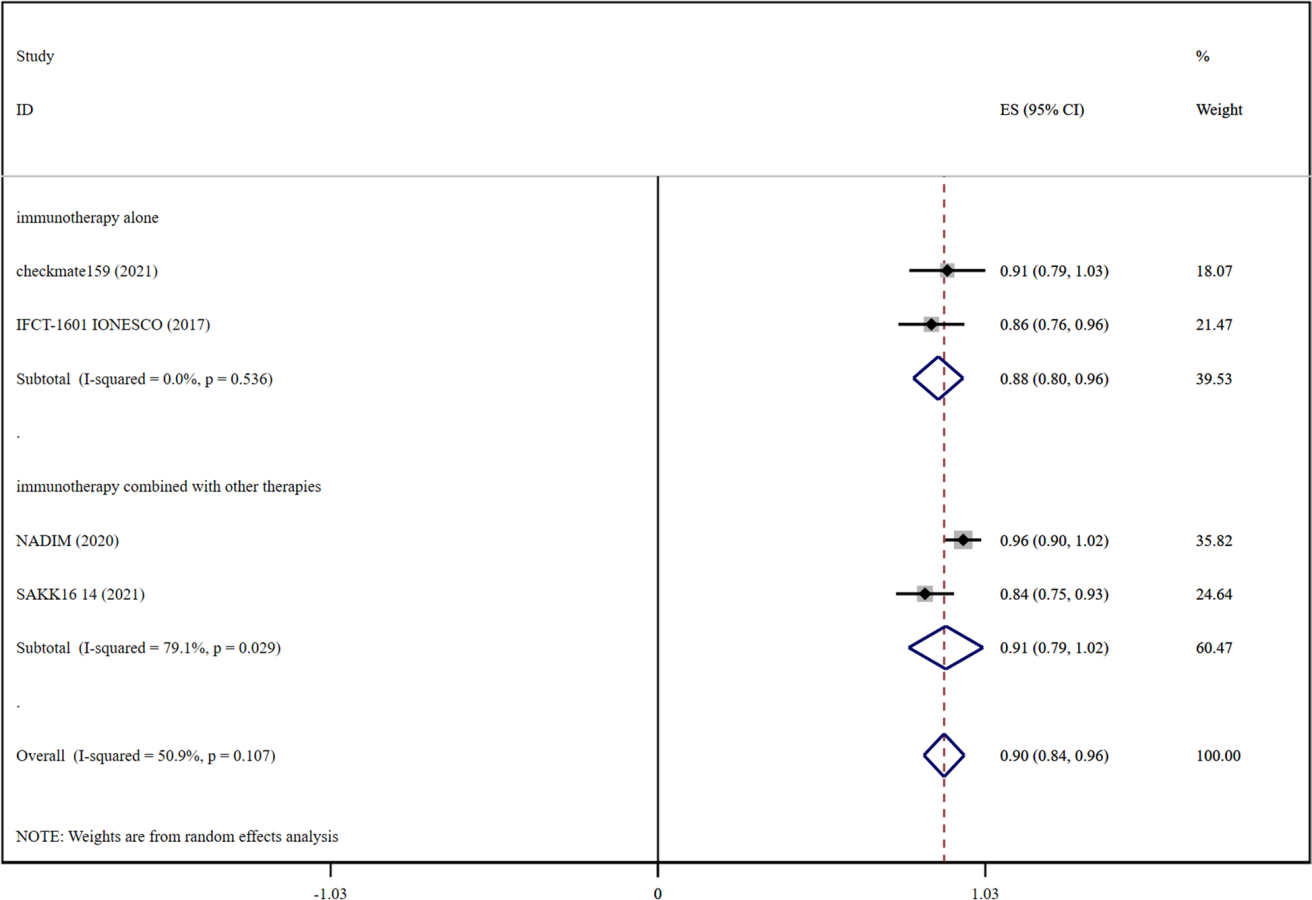
Forest plots presenting with pooled DCR risk ratio analysis in early-stage lung cancer and DCR risk ratio analysis for the cohort of immunotherapy alone and immunotherapy combined with other therapies

**Fig. 4:**
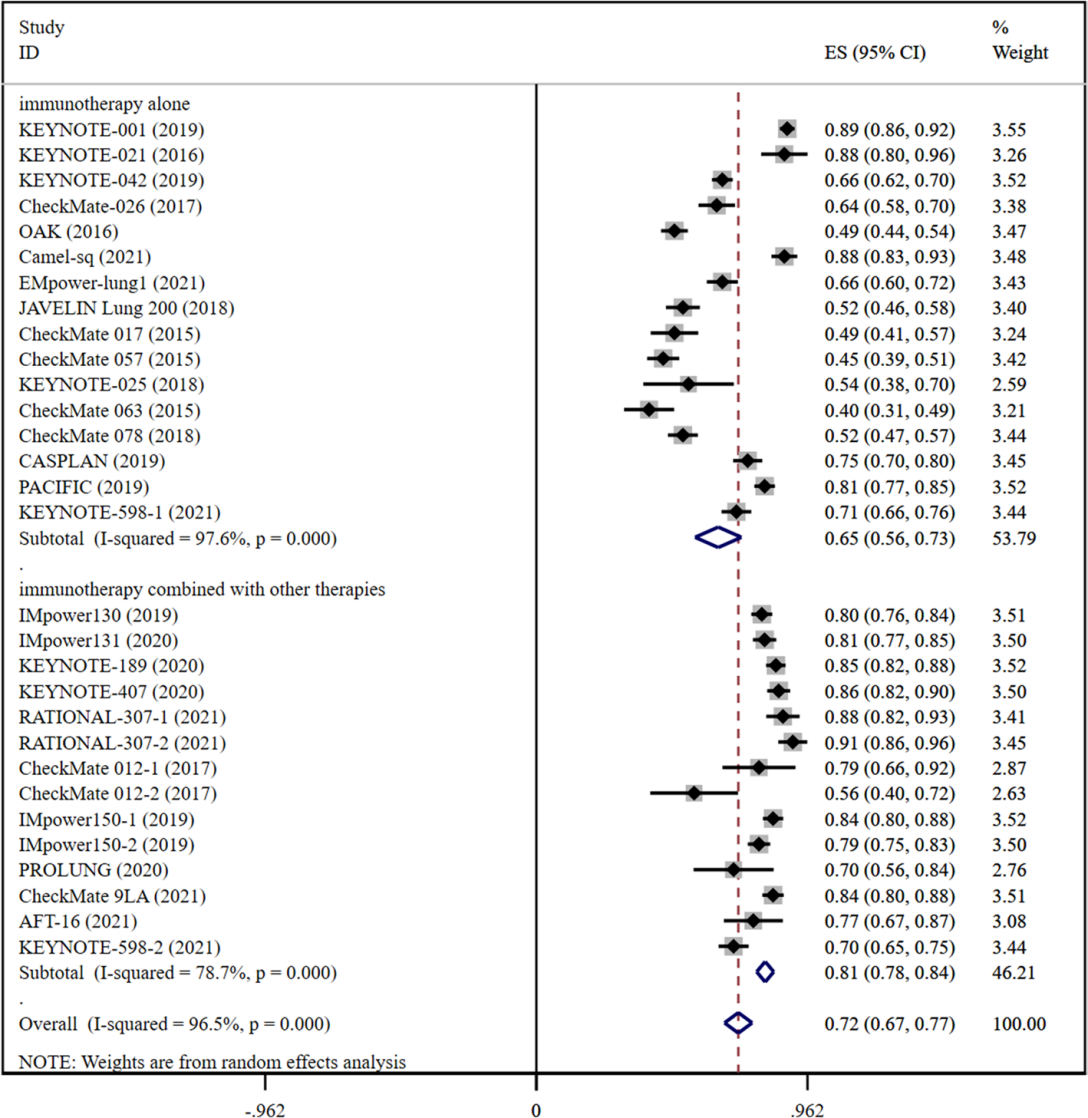
Forest plots presenting pooled DCR risk ratio analysis in advanced lung cancer and DCR risk ratio analysis for the cohort of immunotherapy alone and immunotherapy combined with other therapies

The pooled RR for DCR was recorded to be 0.90 (0.84–0.96) in early-stage lung cancer (Fig. 3) and 0.72 (0.67–0.77) in advanced lung cancer (Fig. 4), which showed that immunotherapy exhibited better efficacy in early-stage lung cancer.

In the immunotherapy alone cohort, immunomonotherapy significantly improved DCR in early-stage lung cancer as compared to advanced lung cancer. The pooled RR for DCR in early-stage lung cancer was recorded to be 0.88 (0.80–0.96) (Fig. 3) and 0.65 (0.56–0.73) in advanced lung cancer (Fig. 4).

In the case of the cohort for a combination of immunotherapy with other therapies, pooled RR for DCR in early-stage lung cancer was 0.91 (0.79–1.02) (Fig. 3), whereas in advanced lung cancer, it was recorded to be 0.81 (0.78–0.84) (Fig. 4). The number of studies on the combined treatment of DCR reported for early lung cancer was only greater than 1, so DCR data for combined treatment of early lung cancer represented the data for the combination of immunotherapy and chemotherapy.

In the case of the cohort for a combination of immunotherapy with chemotherapy, pooled OR for DCR was recorded to be 0.91 (0.79–1.02) in early-stage lung cancer (Fig. 3) and 0.84 (0.81–0.86) for advanced lung cancer (Additional file 1: Fig. S4). According to the results of DCR, the efficacy of the combination of immunotherapy with chemotherapy was found to be better in early-stage lung cancer than in advanced lung cancer.

### Safety

In the case of immunotherapy alone cohort and cohort for a combination of immunotherapy with chemotherapy, the TRAEs and TRAEs of grade 3 or higher were reported to be significantly reduced by the effect of immunotherapy in early-stage lung cancer, when compared with advanced lung cancer.

The pooled TRAEs for patients with early-stage lung cancer was 72%, while the pooled TRAEs for grade ≥ 3 were 28%. In comparison to this, the pooled TRAEs for patients with advanced lung cancer were 80% and the pooled TRAEs for grade ≥ 3 were 41%.

For the immunotherapy alone cohort, TRAEs of grade 3 or higher were recorded to be 28% for patients with early-stage lung cancer and advanced lung cancer. The TRAEs and TRAEs of grade 3 or higher for patients with advanced lung cancer were 74% and 28%, respectively.

In the case of the cohort for a combination of immunotherapy with chemotherapy, the TRAEs and TRAEs of grade 3 or higher for patients with early-stage lung cancer were 93% and 47%, respectively. The TRAEs and TRAEs of grade 3 or higher for patients with advanced lung cancer were 94% and 68%, respectively.

## Discussion

Recent clinical studies, including Camel-sq, RATIONALE 304 [19], and RATIONALE 307 [18], established/confirmed that immunotherapy achieved good/better outcomes in the treatment of advanced lung cancer. The ORR for the immunotherapy group in Camel-sq and RATIONALE 304 was recorded to be 68.4% and 57.4%, respectively. In the same year, clinical studies, like SAKK 16/14 [21], CheckMate 816, and NADIM [26], also showed that neoadjuvant immunotherapy exhibited a higher pathological remission rate and clinical benefit in early resectable lung cancer. However, none of the available studies proved whether immunotherapy incurred a better effect in early lung cancer or advanced lung cancer [46]. In the current systematic review, ORR and DCR were used as evaluation indexes/indices to discuss the efficacy of immunotherapy in early lung cancer and advanced lung cancer. The study also analyzed the safety of immunotherapy in both cases.

According to ORR data for the present analysis, immunotherapy combined with other treatments, especially chemotherapy, appeared to be better in early-stage lung cancer than advanced lung cancer, while immunotherapy alone appeared to be better in advanced lung cancer. In terms of DCR, both immunotherapy alone and immunochemotherapy combined with chemotherapy exhibited better efficacy in early-stage lung cancer than in late lung cancer. In terms of safety, both immunotherapy alone and a combination of immunotherapy with chemotherapy exhibited better safety in early-stage lung cancer than late lung cancer.

Altogether, the current systematic review suggested that immunotherapy, especially in combination with chemotherapy, improved disease response rates in early-stage lung cancer as compared to advanced lung cancer. Besides this, it also exhibited a higher/better safety profile.

Many previous clinical and preclinical evidence suggested that high tumor load incurs a negative impact on anticancer immunity [47, 48]. A recent review article summarized the evidence supporting tumor burden as a biomarker to guide the use of immune checkpoint inhibitors. The study also described the data and provided a perspective on various tools used for the assessment of tumor burden [49]. It has long been debated that ICIs act on the immune system, which is known to be active before the development of tumors. Therefore, an increase in tumor volume is likely to be suggestive of the fact that the immune system is unable to inhibit the growth of the tumor, and in some ways, it is less effective than the immune systems of patients with a lower tumor burden [50]. In addition to this, cancer itself might cause general damage to a patient’s biological functions, including the immune system. With the progression of cancer, the immune system is likely to get deteriorated further. It has been previously shown that the tumor load of early-stage lung cancer is not high, and the immune system is less damaged by the effect of cancer. Therefore, the efficacy of immunotherapy in early-stage lung cancer is generally recorded to be better than advanced lung cancer, and the probability of adverse reactions is also smaller than that for advanced lung cancer [51].

In addition to this, a growing body of clinical evidence supported the synergistic effects of the combination of ICIs with chemotherapy [52], which is consistent with the findings of the present study. In particular, ORR and DCR results for the cohort of a combination of immunotherapy with chemotherapy were better than those reported in the case of immunotherapy alone cohort, both in early-stage and advanced lung cancer. Some studies believe that chemotherapy can not only increase the immunogenicity of tumor cells through a variety of cellular reactions [53] but also eliminate MDSC and regulatory T cells and reduce the immunosuppressive factors in the tumor microenvironment [54]. However, the specific mechanism involved is still unclear.

The present study was associated with certain limitations. The current meta-analysis was dependent on published results instead of individual patient data. Moreover, enough clinical studies are not available on immunotherapy for early lung cancer. In particular, only three clinical studies reported DCR for a combination of immunotherapy with other therapies for early-stage lung cancer, which included two studies on the combination of immunotherapy with chemotherapy and only one study on dual immunotherapy. In the view of a limited number of clinical studies on early-stage lung cancer that reported DCR data for the combination of immunotherapy with other therapies, the related results must be interpreted with caution.

## Conclusion

The findings of the present review highlighted that the benefits of immunotherapy were higher in early-stage lung cancer as compared to advanced lung cancer, especially for the combination of immunotherapy and chemotherapy. Additionally, the safety of immunotherapy, whether alone or in combination with chemotherapy, was recorded to be higher in early-stage lung cancer than in advanced lung cancer.

The results of the study recommended the application of immunotherapy, especially in combination with chemotherapy, for the improvement of survival in patients with early-stage lung cancer. These conclusions of the study need to be confirmed in the future.

## Supplementary Information

**Additional file 1: Fig. S1.** A selection flowchart for the searched articles. **Fig. S2.** Forest plots presenting pooled ORR risk ratio analysis in early-stage lung cancer for the cohort of immunotherapy combined with chemotherapy. **Fig. S3.** Forest plots presenting pooled ORR risk ratio analysis in advanced lung cancer for the cohort of immunotherapy combined with chemotherapy. **Fig. S4.** Forest plots presenting pooled DCR risk ratio analysis in advanced lung cancer for the cohort of immunotherapy combined with chemotherapy.

## Data Availability

All data generated or analyzed during this study are included in this published article (and its supplementary information files).

## Abbreviations

ORR: Objective response rate
DCR: Disease control rate
TRAEs: Treatment-related adverse reactions
NSCLC: Non-small cell lung cancer
CR: Complete response
PR: Partial response
SD: Stable disease
RR: Risk ratio
NR: Not reported
RRs: Risk ratios
