# Supplementary material for: Comparison between immunotherapy efficacy in early non-small cell lung cancer and advanced non-small cell lung cancer: a systematic review
Source: BMC Med. 2022 Nov 7;20:426. doi: 10.1186/s12916-022-02580-1 (PMC9641944; doi:10.1186/s12916-022-02580-1)
Supplement: Supplementary file 1 — Additional file 1: Fig. S1. A selection flowchart for the searched articles. Fig. S2. Forest plots presenting pooled ORR risk ratio analysis in early-stage lung cancer for the cohort of immunotherapy combined with chemotherapy. Fig. S3. Forest plots presenting pooled ORR risk ratio analysis in advanced lung cancer for the cohort of immunotherapy combined with chemotherapy. Fig. S4. Forest plots presenting pooled DCR risk ratio analysis in advanced lung cancer for the cohort of immunotherapy combined with chemotherapy. [file 12916_2022_2580_MOESM1_ESM.docx]

additional file: Supplemental Methods:

Search strategies and number of studies yielded from each database.

Pubmed: 3378 Results

((immunotherapy [Title/Abstract]) OR (PD-1 inhibitor [Title/Abstract]) OR (PD-L1 inhibitor [Title/Abstract]) OR (programmed death 1 inhibitor [Title/Abstract]) OR (programmed death ligand 1 inhibitor [Title/Abstract]) OR (CTLA-4 inhibitor [Title/Abstract]) OR (cytotoxic T-lymphocyte antigen 4 inhibitor [Title/Abstract])) AND (((early lung cancer [Title/Abstract]) OR (early stage of lung cancer [Title/Abstract])) OR ((advanced lung cancer [Title/Abstract]) OR (Late stage lung cancer [Title/Abstract]))) AND ((Randomized Controlled Trial [Title/Abstract]) OR (clinical trial [Title/Abstract]) OR (RCT [Title/Abstract]))

Cochrane: 989 Results

('non small cell lung cancer'/exp OR 'early lung cancer':ab,ti OR 'early stage of lung cancer':ab,ti OR 'advanced lung cancer':ab,ti OR 'Late stage lung cancer':ab,ti) AND ('immunotherapy'/exp OR 'pd-1 inhibitor':ab,ti OR 'pd-l1 inhibitor':ab,ti OR 'programmed death 1 inhibitor':ab,ti OR 'programmed death ligand 1 inhibitor':ab,ti OR 'CTLA-4 inhibitor':ab,ti OR 'cytotoxic T-lymphocyte antigen 4 inhibitor':ab,ti) AND (Randomized Controlled Trial/exp OR clinical trial:ab,ti OR RCT:ab,ti)

Embase: 400 Results

#1 'immunotherapy' OR 'pd 1 inhibitor' OR 'pd-l1 inhibitor' OR 'programmed death 1 inhibitor' OR 'programmed death ligand 1 inhibitor' OR 'ctla-4 inhibitor' OR 'cytotoxic t-lymphocyte antigen 4 inhibitor'

#2 'early lung cancer' OR 'early stage of lung cancer' OR 'advanced lung cancer' OR 'late stage lung cancer'

#3 'Randomized Controlled Trial' OR 'clinical trial' OR 'RCT'

#4 #1 AND #2 AND #3


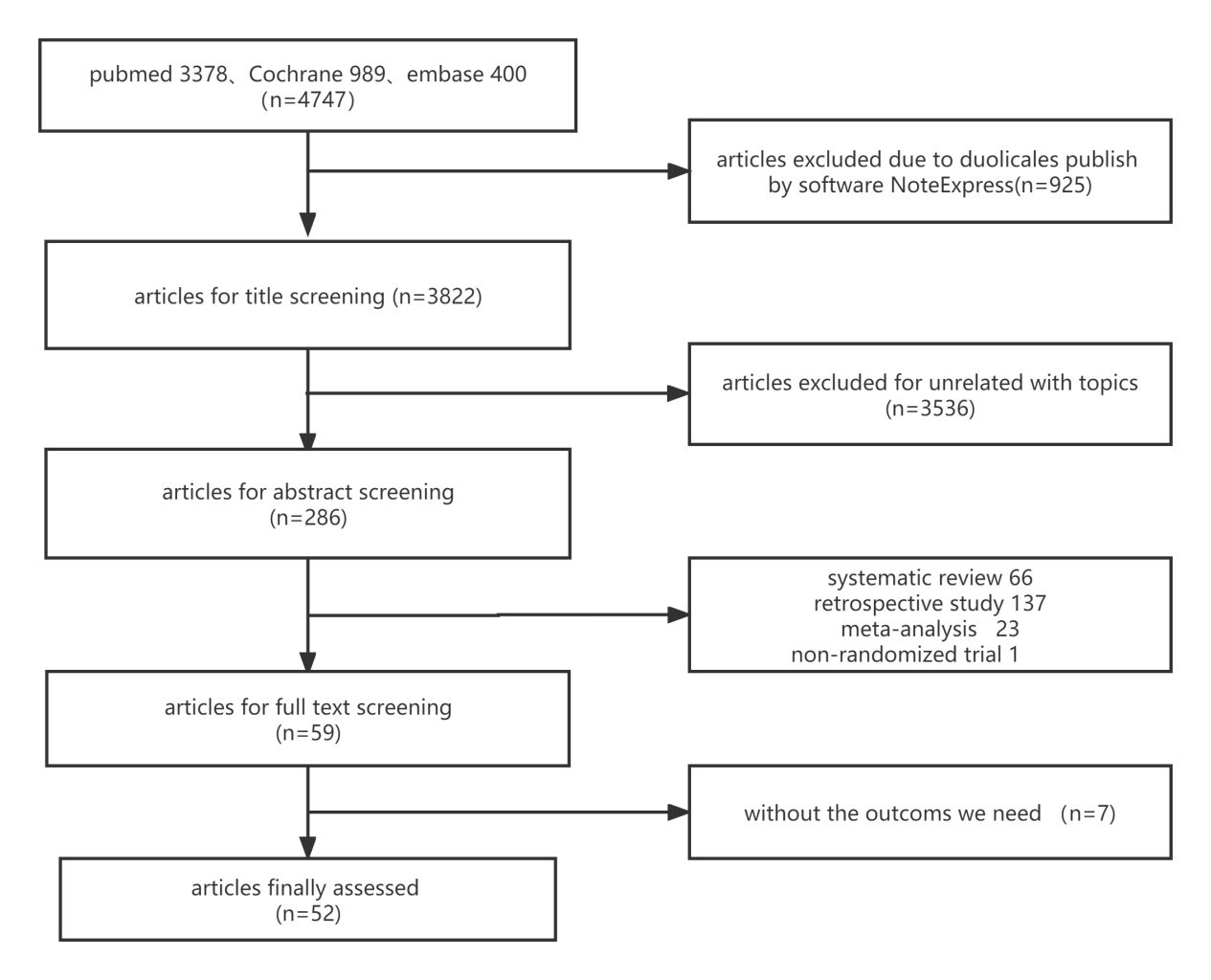


Additional file: Fig. S1: A selection flowchart for the searched articles.


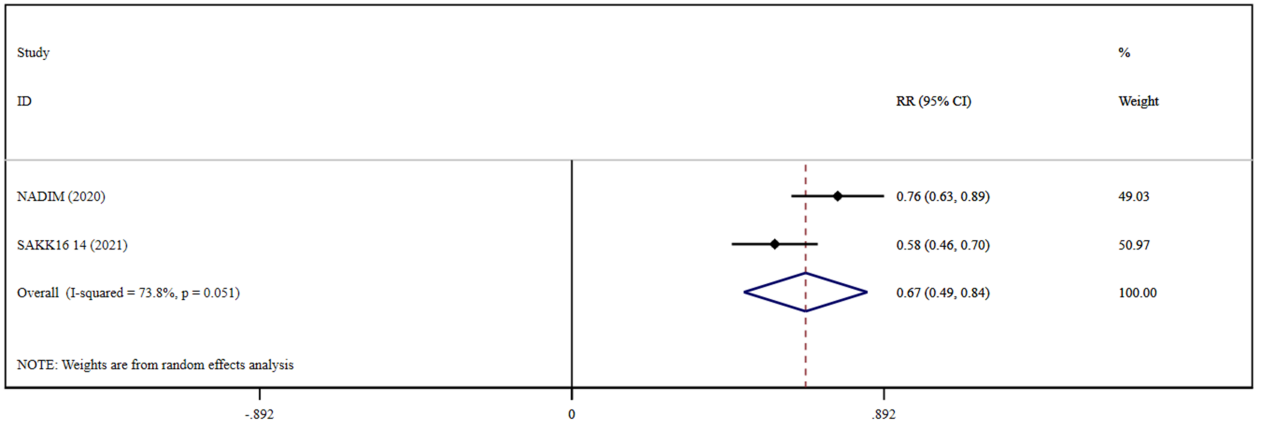


Additional file: Fig. S2: Forest plots presenting pooled ORR risk ratio analysis in early-stage lung cancer for the cohort of immunotherapy combined with chemotherapy.


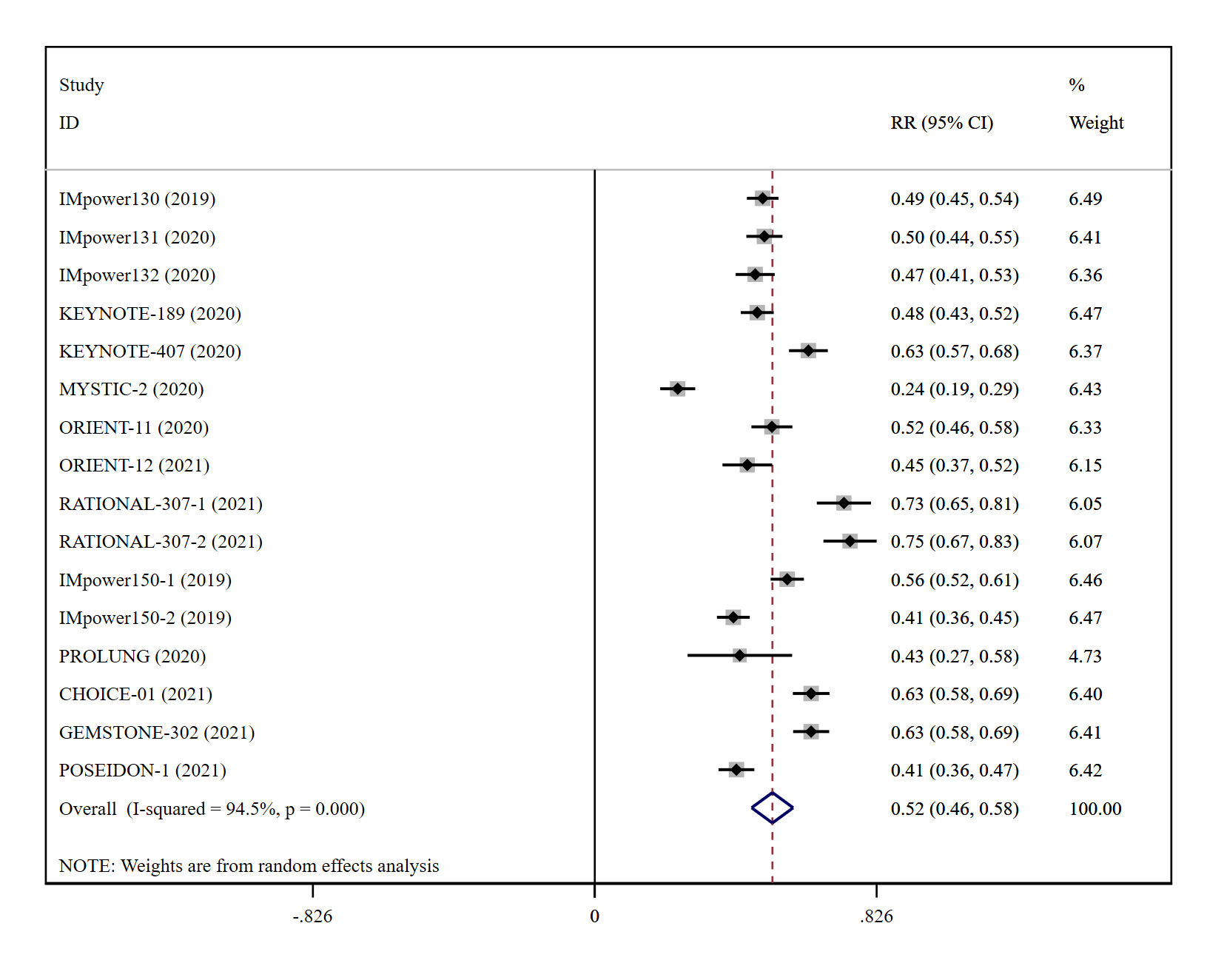


Additional file: Fig. S3: Forest plots presenting pooled ORR risk ratio analysis in advanced lung cancer for the cohort of immunotherapy combined with chemotherapy.


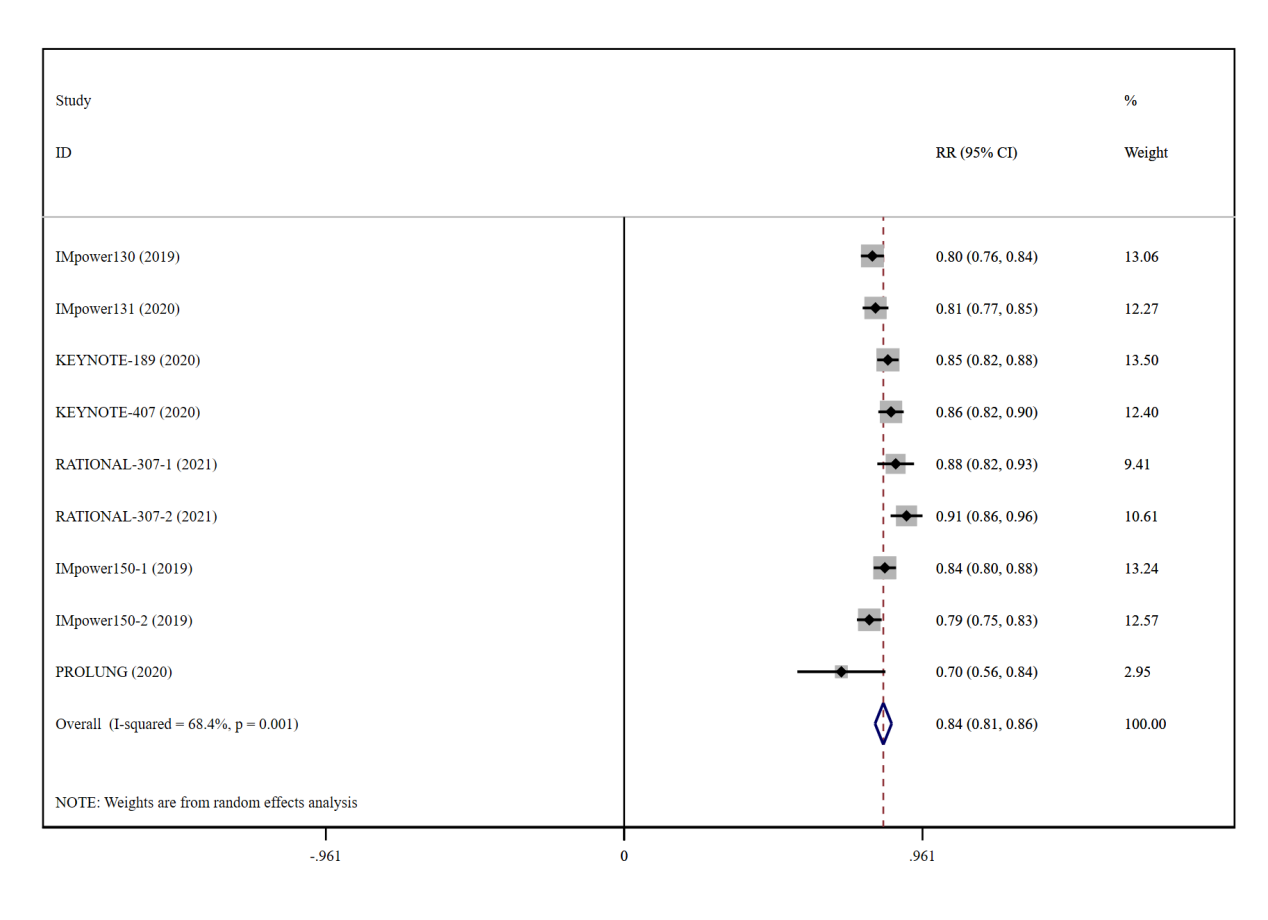


Additional file: Fig. S4: Forest plots presenting pooled DCR risk ratio analysis in advanced lung cancer for the cohort of immunotherapy combined with chemotherapy.
